# Do governance choices matter in health care networks?: an exploratory configuration study of health care networks

**DOI:** 10.1186/1472-6963-13-229

**Published:** 2013-06-24

**Authors:** Annick Willem, Paul Gemmel

**Affiliations:** 1 Faculty of Medicine and Health Sciences, Department of Movement and Sport Sciences, Ghent University, Watersportlaan 2, Ghent 9000, Belgium; 2 Faculty of Economics and Business Administration, Ghent University, Tweekerkenstraat 2, Ghent 9000, Belgium

**Keywords:** Governance, Health care, Networks, Organization theory

## Abstract

**Background:**

Health care networks are widely used and accepted as an organizational form that enables integrated care as well as dealing with complex matters in health care. However, research on the governance of health care networks lags behind. The research aim of our study is to explore the type and importance of governance structure and governance mechanisms for network effectiveness.

**Methods:**

The study has a multiple case study design and covers 22 health care networks. Using a configuration view, combinations of network governance and other network characteristics were studied on the level of the network. Based on interview and questionnaire data, network characteristics were identified and patterns in the data looked for.

**Results:**

Neither a dominant (or optimal) governance structure or mechanism nor a perfect fit among governance and other characteristics were revealed, but a number of characteristics that need further study might be related to effective networks such as the role of governmental agencies, legitimacy, and relational, hierarchical, and contractual governance mechanisms as complementary factors.

**Conclusions:**

Although the results emphasize the situational character of network governance and effectiveness, they give practitioners in the health care sector indications of which factors might be more or less crucial for network effectiveness.

## Background

Health care networks are inter-organizational collaborations among independent health care organizations or individual care professionals. Such networks are widely accepted and used to provide integrated health care services [1]. Provan and Kenis [2] emphasize the need to study the networks as wholes, as entities in themselves rather than as a summation of dyad collaborations between two partners in a network. This entity is neglected as a level of analysis in network research but also as a level of management in practice [2]. Friedman and Goes [3] explain the many hurdles networks face that prevent them from delivering the expected results despite the enormous amounts of financial, human, and clinical resources spent. Given the magnitude of resources involved in health care networks, it is recommended that better care should be taken of the networks themselves. In particular, attention should be paid to governance on the network level, which has turned out to be important in several case studies [4-6]. Furthermore, Rummery [7] cautions about the lack of evidence with respect to the success of health care networks. A number of studies have already contributed to closing the gap in research on how health care networks should be structured and managed; such as Lin [8], van Raak et al. [9], D’Amour et al. [10], and McInnes et al. [11]. While these are important contributions that also emphasize the relevance of organizational aspects in the success of health care networks, they do not focus on the governance part. In a recent study by McInnes et al. [11] stakeholders of clinical networks were interviewed in order to learn about the necessary conditions for effective networks. The network structure, organization and governance, including a large number of sub-themes, were found to be relevant. The work of McInnes et al. [11] reveals the need for more research on conditions for network effectiveness, e.g. on whether networks with an informal structure and governance yield the same results as networks with a more formal form of governance.

In this article, we focus on two governance aspects of health care networks, i.e. governance structure and governance mechanisms, to answer questions such as: “Which kinds of governance exist in health care networks?”, and “Which kind of governance is assumed to be preferable for health care networks?” Provan and Kenis ([12]:231) explain that network governance “involves the use of institutions and structures of authority and collaboration to allocate resources and to coordinate and control joint actions across the network as a whole.” Hence, network governance is about: 1) structures for collaboration (governance structure), and 2) coordination in networks (governance mechanism) [13]. There are three generic types of governance structure, namely the participant-governed network, the lead organization form, and the network-administrative organization [12]. Governance mechanisms refer to the mechanisms used in the network to co-ordinate tasks. There are also three types of governance mechanisms: markets, hierarchy, and relational governance [14,15]. The literature [12] suggests that it is not the choice of governance mechanisms or structures in itself that affects network effectiveness, but the fit of governance mechanisms with governance structure. Governance structure and governance mechanisms are related and interdependent, and can be seen as two dimensions of network governance that can exist in several combinations. The structure, for example, influences the possibilities for effectively using certain governance mechanisms [12].

## Methods

### Setting

A Belgian study on the evolution of health care in Flanders [16] teaches us that networks can be found in many areas of health care, such as psychiatric care, palliative care, oncology, etc., where several care organizations cooperate to treat a particular group of patients; or among hospitals that co-operate for purposes of rationalization. The reasons for collaborating in health care networks in Flanders are: financial pressure, government regulations, sharing scarce human resources, as an alternative to a merger, and to provide patient-centered integrated care. In Flanders, the number of networks boomed in the last decade [16]. The government of Flanders, a region of Belgium, urged the development of health care networks in all areas of health care, and in particular in psychiatric care, because of the complexity of this health care area. Psychiatric care requires cooperation among several organizations in a psychiatric care landscape that is very dispersed. Our study applies to governance in any health care network setting regardless of the specific area of health care. Hence, we opted intentionally for maximum variation in our study of health care networks.

### Research design

A case study research strategy was chosen to study a) if the three kinds of governance mechanisms can be found either in isolation or in combination in networks, b) whether governance structure is associated with a particular governance mechanism, and c) whether there are effective configurations of governance structure, governance mechanisms, and network attributes. Such a method is especially useful when a consistent theory is lacking and the theory needs to be further developed [17], as is the case for the theory on health care network governance. The cases concerned health care networks, and the unit of analysis was the whole network. We opted for a multiple case study setting allowing us to seek patterns and similarities in network characteristics and governance among the different cases. According to Yin [18], case study research is not limited to qualitative research. Case study research can fit within a qualitative or quantitative research strategy or a combination of the two, and such a combination allows triangulation. In each of our cases, qualitative and quantitative data were combined.

### Framework

To answer our study questions, a configuration view on health care networks was chosen. Configuration theory explains organizational effectiveness by means of a variety of interrelated attributes of the organization [19]. “Configuration theory aims at capturing the complexity of organizations and to expose their inner coherent logic ([19]:68)”. Lamothe and Dufour [19] have dealt with the critics of simplistic reductionism in configuration theory by explaining that configuration theory is aimed at explaining the complexity and interdependency of organizational parts and attributes. According to this theory, several combinations of attributes or configurations can exist, but some configurations will be more effective than others. Here, configuration theory is used as a lens to analyze whether combinations of governance choices fit together to result in higher network effectiveness. The theory on network governance and effectiveness has given indications of governance characteristics that are preferable or that fit together, but has not provided empirical evidence [12,20,21]. A large and greatly undefined number of network attributes play a role in network effectiveness. We limit these to the ones that have been mentioned in Provan and Kenis [12] and that form a bridge between governance structure and mechanisms, namely trust, legitimacy, flexibility, and size.

Using the configuration lens, three assumptions on network governance and effectiveness were empirically studied. Before explaining this further, we will elaborate on network effectiveness, which is clearly a tricky subject. Many researchers are struggling with the issue of network performance and, in particular, network effectiveness in a public and nonprofit context [11,22,23]. There is effectiveness on different levels, namely the organizations in the network, the network as a whole, and society [24]. Even when limiting network performance to network effectiveness—defined as reaching the network’s goals—it remains a complex issue because often those goals are unclear or vague and perceived differently by the different partners in the network. Even so, perceived effectiveness can be used as a proxy for a minimum level of network success. For any organization or collaboration, a goal or purpose of the collaboration is implicitly or explicitly present. Effectiveness can therefore be judged by the extent to which network members perceive the network objective to have been reached.

A first assumption of our study is that in a health care context where the government or external stakeholders urge the development of health care networks [25,26], the three kinds of governance mechanisms can be found in networks either in isolation or in combination. Empirical evidence is lacking for the claim that the presence of contractual or hierarchical governance mechanisms in combination or in the absence of relational governance influences network effectiveness. As mentioned before, there are three types of governance mechanisms: markets, hierarchy, and relational governance. The latter is typically associated with networks [14,15]. Relational governance refers to co-ordination based on trust, reciprocity, and common norms and values that are embedded in the relationships between the partners in networks [14,15,27-29]. Markets are governed by contracts and pricing mechanisms [30]. In the context of collaboration in health care, this mechanism refers to the extent to which the collaboration is detailed and formalized in contracts; and here it is labeled as the contractual governance mechanism. In hierarchies, formal authority is the dominant mechanism to co-ordinate and control tasks. In networks, collaboration can be coordinated by hierarchical relations in the network, in which one partner or representative has authority over others; and this is here labeled as the hierarchical governance mechanism. Governance mechanisms in health care networks are, therefore, based on hierarchical, contractual, or relational governance, or a combination of these three. Although relational governance is traditionally thought to be the primary governance mechanism in networks [20,29], the literature suggests that this might not always be the case, at least not in health care organizations [31].

Our second assumption is that, although networks can be governed by means of different types of governance mechanisms, the governance structure is associated with a particular governance mechanism that will be more suited in a certain governance structure. The first type of governance structure, participant-governed network structure, has no separate management entity; and each of the organizations in the network is responsible for decision-making and managing the network. In the participant-governed network structure, trust is important and network governance is dominated by the relational governance mechanism. The second form, that of the lead organization, is characterized by a dominant organization in the network taking responsibility for managing the network. In the third form, the network -administrative organization, a separate entity is created to manage the network. This separate entity is autonomous from the partner organizations in the network and has its own staff. In the lead organization and network-administrative organization forms, organizations in the network give up part or all decision-making power to the lead organization or the administrative entity, respectively. These leading organizations or entities develop some authority over the other organizations, and a kind of hierarchy might be established, resulting in the application of hierarchical governance in the network [12].

Our third assumption is that certain combinations of governance structure, governance mechanisms, and network attributes lead to a more effective network configuration. Kenis and Provan [12,23] mention that the governance form “network-administrative organization” fits best with medium levels of trust, medium to large network size, low levels of flexibility, and medium levels of internal and external legitimacy. Similar indications are made for the participant-governed form and the lead organization form. Herranz [20] links governance mechanisms with network goals in a study on workforce development networks in Boston. Hierarchical governance, for instance, would be more suitable for achieving a stable network with high levels of accountability and a focus on the compliance with regulatory requirements. Relational governance is more suitable for networks that aim at developing trust and establishing or intensifying collaboration. Market or contract based governance is better for networks with innovation and financial objectives [20]. Legitimacy and size might be related to hierarchical governance and to lead organization and network-administrative organization governance structures. The age of the network is included as a control variable because networks might differ in the different stages of their life-cycles [21,23]. Finally, because of the intensive involvement of governmental agencies in health care in Europe, government involvement in the networks was also included. For instance, Milward and Provan [32] explain that in networks in which the government participates to provide services, such as mental health services, a hierarchical relationship might arise between the funding and controlling governmental agencies and the nonprofit organizations providing the services. The governance structure of the network can be deliberately chosen to give certain partners in the network more or less power, e.g. granting sufficient power to the financing government.

### Study subjects

To distinguish between networks and other kinds of collaboration, we used the definition of Provan and Kenis [12:231] i.e. “groups of three or more legally autonomous organizations that work together to achieve not only their own goals but also a collective goal.” Hence, applied to health care networks, any collaboration among at least three autonomous or independent health care providers (e.g. hospitals, residential elderly care, home care organizations, mental health care centers) for which a common goal is defined, fits our definition. This involves a broad range of health care networks. Lists of health care networks to serve as sample frames did not exist, and, therefore, a random sampling was impossible. The networks were, therefore, found and selected by part-time master students, who worked part-time in health care organizations (mainly hospitals). They contacted a colleague who was involved in a health care network that met our definition. Through this snowball sampling method, we were able to select a diverse sample and, although not representative for the total health care network population, there are no assumptions of selection bias that might be related to our topic of study. Our sample consisted of 22 health care networks in Flanders. Most networks were related to psychiatric care either in care institutions or home care situations. Other networks were related to care for the disabled, the elderly, or palliative care. The overrepresentation of mental health care networks had to do with the fact that networks are very popular in this area of health care in Flanders.

### Data collection

Data were collected through questionnaires and semi-closed interviews. One responsible person per network (this is the network co-ordinator, manager, or official representative of the network) was interviewed. Questionnaires were sent to a representative of each organization in the network. Students selecting the networks also assisted in the data collection, but the coding of the data and data analysis was done by the first author. The number of questionnaires per network ranged from 3 to 31, depending on the size of the network and the response rate. The response rate was on average 68 per cent.

In the questionnaires, Likert-scaled items measured the three governance mechanisms (relational governance, contractual governance, and hierarchical governance); four other network characteristics (trust, flexibility, internal and external legitimacy); and network effectiveness. Relational and contractual governance was measured using the scales of Berbée et al. [33]. Hierarchical governance and trust scales were respectively based on Cunningham and Rivera [34] and McAllister [35]. Flexibility and legitimacy scales were developed based on the theory of Provan and Kenis [12]. Legitimacy was divided into internal legitimacy (or the legitimacy of the network for the partners in the network), and external legitimacy (or the legitimacy of the network towards external stakeholders). In the interviews, questions were asked about the governance structure of the network, the sector, objectives and goals, size and age of the network, government involvement, and whether the network goals had been reached. Network effectiveness was operationalized as perceptions on whether the network was reaching the common network goals. Goals vary among the networks given the diverse sample of networks, which prevented us from using more exact effectiveness measurements. Network effectiveness was measured in the interviews and in the questionnaires by asking questions on how successful the network was in more objective (objectives reached) and subjective (perceived success) terms.

### Data analysis

Following the logic of qualitative comparative analysis, we searched for patterns in the data and conditions for effective networks related to the governance choices. First, the recoding of the questionnaire data was done to transform the data into conditions that could either be present or absent. Table 1 lists the descriptive statistics of the questionnaire data before re-coding. The questionnaire data were used to describe the characteristics of the 22 networks. The questionnaire data resulted in scores between zero and five for each variable and respondent. These data were aggregated per network. Scores per variable on the network level were then re-coded in three categories (low, medium, and high) or in two categories (present or absent).

**Table 1:** Descriptive statistics for the questionnaire data

	**Mean**	**Median**	**Standard deviation**	**Variance**
Relational governance	3.8202	3.7708	0.2335	0.055
Contractual governance	3.0644	3.2187	0.5102	0.2600
Hierarchical governance	3.1407	3.1578	0.3894	0.1520
Affect-based trust	3.7151	3.6138	0.3367	0.1130
Cognition-based trust	3.8952	3.8646	0.2688	0.0720
Internal legitimacy	3.9105	3.8905	0.2690	0.0720
External legitimacy	3.5690	3.4833	0.2889	0.0830
Network flexibility	3.3486	3.3250	0.4007	0.1610
Effectiveness	3.7856	3.7381	0.3546	0.1260

The interview data were transcribed and also coded. To code governance structure into network-administrative organization, participant-governed network, and lead organization, the criteria of Provan and Kenis [12] for these three types were used. Other interview data were used to code the subsector (psychiatric care, elderly care, palliative care, disabled care, other), the size (large indicating 10 or more partners in the network, small indicating less than 10 partners in the network), age (young indicating that it had existed for less than six years, old indicating an existence of at least six years), and government involvement (partner, financing, no important role).

Networks were scored as either effective or ineffective based on a combination of the score on the aggregated questionnaire data and the statements made in the interviews. As a result there is a score, per network, for network effectiveness obtained through the interviews with a representative per network, and a score obtained through aggregating the scores for network effectiveness per respondent of the organizations in the network. These two scores were expressed as low, medium, high. Networks were indicated as effective if they scored respectively high twice, or high and medium once each on the interview and questionnaire data.

Second, after re-coding, data were presented as conditions. For each network, we presented information on the goal, network characteristics, network governance, and effectiveness; reducing the case information to combinations of attributes. This followed the logic of qualitative comparative analysis and enabled analyses on small samples with a combination of quantitative and qualitative data [36]. Results for the first two assumptions were derived from the descriptive data. The third assumption on configurations required the search for configuration in the data and the development of a truth table in which raw data were transferred as a list of configurations for each case, with configurations as combinations of conditions and an outcome [37].

### Ethics

The study obtained approval from the independent Medical Ethics Committee of Ghent University Hospital. Participation in this study was voluntary and anonymous reporting was guaranteed. We use here a pseudonym for the names of the networks to guarantee this anonymity, which was necessary to receive sufficient cooperation for our study. In some service areas there were only a few active networks. Hence, we were also careful not to disclose network identity in the description of the goals of the network. Informed consent was obtained from the respondents. A letter accompanied the questionnaires and interviews to inform the respondents of the objective of the study and their freedom to participate or withdraw from the study. Each respondent was individually approached by the researchers and asked for participation to avoid that some respondents would feel obliged to participate because of an agreement to participate between the researchers and the network.

## Results

Table 2 provides an overview of the characteristics of the networks and relative indications of the scores on a number of variables in the form of a truth table. Note that all indications of high and low or strong and weak are relative within the sample. For instance, “high levels of trust” refers to a network in which the score on the trust variable is clearly higher than the average score on the trust variable in the sample of networks. For the qualitative comparative analysis, data in the truth table were coded with 0 or 1, or 0, 1 or 2, but to increase readability we used labels instead of numeric values. The correlation analysis of the questionnaire data revealed a high correlation (.67) between cognition and affect-based trust. Therefore, both trust dimensions were combined as one condition in the table.

**Table 2:** Network characteristics

**Name**	**Objective**	**S **^**1**^	**A**^**1**^	**G**^**1**^	**F**^**1,2**^	**IL**^**1,2**^	**EL**^**1,2**^	**T**^**1,2**^	**GS**^**1**^	**RG**^**1,2**^	**CG**^**1,3**^	**HG**^**1,3**^	**E**^**1,3**^
drug abuse	Optimizing cooperation among care providers in the network	L	O	F	L	M	M	L	P	M	Y	Y	1
hospital cooperation	High quality care, economic efficiency and geographical accessibility	S	Y	N	M	M	M	M	P	H	Y	Y	1
psychiatric care 1	Increasing quality of care services for the patients, increasing professionalism among the partners	S	Y	F	H	H	H	H	P	H	Y	Y	1
psychiatric care 2	Providing after-service care and patient allocation	S	O	N	M	M	M	H	P	M	Y	Y	1
palliative care 1	Providing a full range of services	L	O	F	H	H	H	M	N	M	Y	Y	1
psychiatric home care 2	Promoting a specific action in all hospitals	L	O	F	M	M	M	M	P	M	Y	Y	1
complex psychiatric care 1	Developing cooperation	L	O	F	M	M	H	M	L	M	Y	Y	0
hospital network	Support to care providers, co-ordination and consultation	L	Y	P	M	M	M	L	N	L	N	Y	0
integrated care and welfare	Support to care providers, co-ordination	L	O	P	M	M	M	M	P	H	N	N	0
psychiatric care 3	Increasing the possibilities for patients to live independent	L	Y	N	M	M	H	M	P	M	Y	Y	0
psychiatric care 4	Offering optimal quality of care	S	Y	F	M	L	M	L	P	L	Y	Y	0
psychiatric home care 1	Providing services to partners allowing a human, innovative, and professional care for elderly	L	O	F	H	M	M	M	P	M	Y	Y	0
disabled care 1	Consultation and data gathering	S	Y	P/F	H	M	L	H	P	M	Y	Y	0
elderly care1	Developing residential care in a region as requested by the regional policy makers	S	O	N	M	M	M	H	P	H	Y	Y	0
psychiatric care 5	Improving care	S	O	F	M	M	M	M	P	M	Y	Y	0
elderly care 2	Improving consultation, co-ordination; providing integrated care; increasing knowledge of target patient groups	S	Y	P	M	H	M	M	P	M	N	N	0
psychiatric care 6	Providing individualized support to disabled	L	O	F	M	M	M	M	P	M	N	N	0
complex psychiatric care 2	Maintaining continuity, and quality of care, providing training, advice, support, and a registration system to care providers	S	Y	F	L	L	M	L	L	L	Y	Y	0
disabled care 2	Developing good integrated care	L	O	N	M	M	L	M	P	M	Y	Y	0
psychiatric care 7	Optimizing palliative care through information, and training	L	Y	F	L	L	L	M	P	L	Y	Y	0
palliative care 2	Increasing the possibilities for patients to live independent through long term professional and systematic care provision	L	O	F	M	M	M	M	P	M	Y	Y	0
palliative care 3	Providing information; co-ordination, training, advice, logistic support, and evaluation to care providers and volunteers	L	O	F	M	M	M	M	N	M	Y	Y	0

^1^*S* size ( size S < 10 organizations, size L > 9 organizations); *A* age (age Y < 6 years, age O > 5 years); *G* government involvement (*P* Partner, *F* Financing, *N* No large impact);

*F* Flexibility, *IL* Internal legitimacy, *EL* external legitimacy, *T* Trust, *GS* Governance Structure (*P* participant-governed network, *L* lead organization network, *N* network administrative organization); *RG* Relational Governance, *CG* Contractual Governance, *HG* Hierarchical Governance, *E* Effectiveness,

^2^High, mediate or low levels of this variables; ^3^Yes (present) or No (not present).

Based on the data, the following observations were made. Six out of the 22 cases were classified as effective. Qualitative data revealed that goals differ, even within a similar category of care. For instance, “Psychiatric care networks 1 and 2” were both perceived as effective, but the first network’s main objective was improving care while the second network’s was developing additional care. “Psychiatric care network 2” did not fully reach its goals, although it was still perceived as effective. Objectives in the networks ranged from cooperation and patient referral to integrated care and establishing new services. However, easy to reach objectives did not per se lead to high effectiveness. For instance, the objective of the “hospital network” was modest in terms of the required intensity of collaboration and efforts, but was still not particularly successful. Different kinds of health care services were involved, but differences in network characteristics were not clearly related to differences in care. The lead organization governance structure was found only twice, each time in psychiatric care. The network administration organization form was found three times of which two were present in palliative care. The government was present in most of the health care networks, mainly as a funding and controlling institution. The government was most often the funding body (9 out of 11 times) in the psychiatric care networks, and never a partner. Most networks had the participant-governed network form (17/22). The network-administrative organization form was preferred in some of the larger networks. Even though a manager or management system was often established at network level, most networks could still be considered as having a participant-governed form because decision-making and responsibilities remained shared among the partners. Levels of trust, internal and external legitimacy, and flexibility varied but were not systematically related to a particular governance mechanism or structure.

Related to the first assumption on the presence of governance mechanisms, the data indicated that relational governance was supplemented with either hierarchical or contractual governance, or both, in 19 of the 22 cases. In 15 of the 22 cases, relational, contractual, and hierarchical governance were all three combined. One network was even dominantly governed through hierarchy, and three others by a combination of hierarchical and contractual governance in the absence of relational governance. Hence, related to our first assumption, we can state that the relational governance mechanism was indeed not the dominant governance mechanism in our sample of networks. All combinations of governance mechanisms seemed to be present. Additional qualitative interview data showed further that hierarchical governance was mainly put in practice through procedures and a coordinator/manager on the network level. Co-ordinators, network managers, procedures, and contracts were also often part of the funding requirements.

Our second assumption suggests a relationship between governance mechanisms and governance structure. Indeed, the combination of a participant-governed network and relational governance was present in 15 of the 22 cases; and lead organization and network-administrative organization networks were combined with hierarchical coordination. However, hierarchical governance was not exclusive for the lead organization and network-administrative organization networks.

A third assumption on configurations suggests that combinations of conditions result in higher network effectiveness. Combinations of all conditions resulting in effective or ineffective networks could not be identified. However, subsets of conditions could be related to less effective cases. For instance, combinations of larger and younger networks were only found among the less effective cases. A combination of low levels of relational governance and trust were related to non-effective cases. The clearest result was found for the combination of the three governance mechanisms. Among the less effective networks, several did not use all three types of governance mechanisms. The absence of either contractual, relational, hierarchical or two of these was found only among the less effective cases. Other conditions also occurred several times among the less effective cases, while these conditions were absent among the more effective ones. Low levels of internal and external legitimacy occurred only among the less effective networks. Trust and flexibility indicated contradictions, because these were found among both the more and less effective networks, e.g. the drug abuse network had low trust and flexibility. All four networks in which the government was not only the regulator or the financing body but also a partner were among the less effective ones.

## Discussion

Herranz [20] warns that networks might be managed as hierarchies in a way that is hardly different from traditional administration. This is not shown in our data, because in most networks two or three governance mechanisms were combined. Contractual or hierarchical governance was not particularly used to compensate for low relational governance or, for instance, for low levels of trust. Based on our data, we can state that in health care settings, networks do not limit governance mechanisms to relational governance. The networks seem to deal with the complexity of care by combining the three governance mechanisms. The literature on collaborative networks in the public sector already indicated that in practice hierarchy and relational governance are jointly and unavoidably present in networks. Lowndes and Skelcher [13], for instance, found in a study of UK urban regeneration partnerships that the three governance mechanisms (contracts, hierarchy, and relational) can exist in network forms of inter-organizational collaboration, and that these modes can co-exist and depend largely on the stage in the life-cycle of the network organization. Bode and Firbank [31] studied home-care networks for the elderly in various countries and found that such networks can have very different governance mechanisms, ranging from more bureaucratic to more market-oriented. Berbée et al. [33] distinguish between contractual and relational governance mechanisms in health care exchange relationships and found in a Belgian health care institute that contractual and relational governance reinforce each other rather than be conflicting. However, a balanced combination of governance mechanisms as a condition for network effectiveness had not yet been suggested, nor studied. Our data gave indications of the importance of a balanced combination of relational, contractual, and hierarchical governance. The most effective networks in our sample had such a balanced combination; while in seven out of 16 cases of the less effective networks such a balance was absence. The literature [27] has focused on tensions among governance mechanisms, but from a control perspective, especially in a complex health care environment, a balanced combination of governance mechanisms might increase rather than reduce network effectiveness [33]. Hence, our study does not confirm the findings of Entwistle et al. [27], who point at hierarchy and contracts as sources of dysfunctions in networks. The presence of hierarchical governance might only be ineffective if hierarchy is the only dominating governance mechanism. In our data, the presence of hierarchical governance in combination with low levels of relational governance was found among the less effective networks.

The most frequent governance structure was the participant-governed network. Based on a systematic literature review, Jackson et al. [38] found that the participant-governed and network-administrative organization types were both present in multiple health care partnerships. In our sample, only in situations in which networks became very large (e.g. include more than 30 organizations) did a network-administrative organization take over governance tasks. In two cases of psychiatric care, networks were governed by a lead partner. This was mainly due to some historical evolutions or larger resources of one partner. Although decision-making was then concentrated in the network-administrative organization form or in one partner, these networks were not perceived as more hierarchical. The literature suggests that governance mechanisms match governance structure [12]. Although participant governance was the dominant structure, it was not systematically dominated by relational governance. However, only in two cases did governance structure and governance mechanism clearly contradict each other, namely through a combination of a participant-governed network and low relational governance. Furthermore, Provan and Kenis [12] suggest that in participant-governed networks, internal legitimacy is more easily established than is external legitimacy [12]. In our sample, participant-governed structures did not guarantee high internal legitimacy and did not per se result in low external legitimacy; nor did these networks tend more towards flexibility.

The results indicate that networks differed considerably in health care services. The differences were found in all characteristics, such as governance structure and mechanisms, level of legitimacy, trust, objectives, government involvement, age, and size. Differences could not be explained only by the variations in type of health care provided. Hence, we cannot conclude that there is a dominant network type for a certain type of care or that there should be a certain type, because networks that were very different in terms of the studied characteristics can be equally effective. Hence, our data confirmed that there were no indications of one superior governance type or mechanism. However, there were indications that certain characteristics are less favorable and might cause or be an indication for less successful networks. Note that each of these characteristics are not necessary nor sufficient conditions for network ineffectiveness. Relevant characteristics were: government agencies as partner, low levels of internal and/or external validity, low levels of relational governance, and relational governance that is not combined with hierarchical and contractual governance mechanisms.

The role of governmental agencies in networks clearly needs further exploration. McGuire and Agranoff [39] have already indicated the difficult new role governmental agents need to play when entering networks as partners instead of as financing, regulating, or controlling bodies. The autonomy of health care professionals might be threatened when governments participate [40], which might cause lower network effectiveness.

A clear life-cycle effect could not be observed. In other words, no clear differences between younger and older networks were observed. An exception were the networks that were young and large, and were also less effective. An explanation might be that new networks that are large from the beginning need time to establish trust, legitimacy, governance mechanisms and are, therefore, less effective in the first stage of their life-cycle. Furthermore, we expect that the life-cycle might differ for each network. Respondents of networks that have existed one to three years mentioned that collaboration was still developing, while respondents of networks of more than 10 years also felt that the network was still in a developmental phase. Other networks of the same age (either less than three or more than 10 years) were in a more mature phase. As Provan et al. [4] show, networks evolve due to changes in the environment, but the change pattern might be unique for each network.

Our study is clearly of a very exploratory nature, and further qualitative and quantitative research is needed. This is necessary to test the assumed relationships in our study further, but also to explore which other network characteristics and network management issues might be crucial. Our study is especially useful to indicate paths for further analysis, such as the role of the government and the complementarity of governance mechanisms, as well as paths that might be less fruitful for further research, such as flexibility, age and size. The results are based on a diverse sample of only 22 networks. We specifically aimed for this diversity in order to see whether patterns could be observed in health care networks in general, but the diversity may have blurred our results. Further research in a larger and more homogenous sample is recommended. Such a homogenous sample would enable a more precise measurement of effectiveness in terms of outcomes, such as patient satisfaction, or quality of care. The current sample is broad and diverse, and includes a bias towards mental health networks, and is, therefore, not specific for a particular subsector, nor representative for the whole population of health care networks. On the other hand, the fact that psychiatric care networks did not differ from the other networks might also be an indication that the conditions or characteristics are not that sector-specific. Finally, to study our three assumptions and to combine qualitative and quantitative data, the data were reduced to conditions and effectiveness was coded in a binary way. This is a simplification inherent to the method, but excludes richer data on the history and environment of the networks. The binary coding of perceived effectiveness categorizing networks as either effective or ineffective holds two important limitations. First, there might be several levels of effectiveness and by using only two levels, our analysis is very rough. Second, perceptions of effectiveness do not correlate perfectly with directly measurable effectiveness criteria.

## Conclusions

There is clearly a trend towards using more collaborative settings and networks for delivering care. Practice has not waited for theory in terms of evidence on whether and how networks should be developed. This paper is an attempt to describe the networks in terms of governance structures and mechanisms, to learn which type of governance is used and whether we could find indications of a preferred type of governance. This can help practitioners in identifying the dysfunctions in network settings that prevent networks from reaching their objectives. Our study resulted in several findings that are relevant for practice. First, there is a variety of networks based on governance and network characteristics, and there seems to be neither an ideal network form in health care nor different types fitting different types of care. Moreover, several of the relationships or presumed preferred types based on theoretical grounds should not (yet) be considered as rules for network governance before further testing. Second, not only is there neither a dominant nor an optimal network type, there is also not one or more sets of dominant configurations. Therefore, specific combinations of network characteristics cannot yet be taken as guidelines for developing a successful network. Third, relational, contractual, and hierarchical governance mechanisms seem to be complementary factors, not substitutes or conflicting mechanisms. Hence, although guidelines based on our exploratory study are limited, one piece of advice is that a balance in governance mechanisms might be preferable. Hierarchical governance mechanisms in particular might, unexpectedly, be important for the success of health care networks if combined with relational and contractual governance mechanisms. Fourth, networks can be perceived as effective despite lack of trust, or inflexibility; but some characteristics, such as low levels of legitimacy or of relational governance, seem to be occurring more often among the less effective networks. These characteristics, of course, need further testing on a larger scale to see whether there is indeed a statistically significant relationship between them and network effectiveness. Nonetheless, practitioners might look into those variables when searching for causes of underperforming networks. Finally, the major involvement of government agencies in networks requires special attention because these agencies play another role than do most partners.

## Competing interests

This research was not funded by an external organization. The authors declare that they have no competing interests.

The authors declare that they have no relationship with, or financial interest in, any organization pertaining to this article; nor did they receive funding for this research.

## Authors’ contributions

A.W. has been in charge of the literature search, data collection and data analyses, has developed the main arguments presented in the article, has led initial drafting of the article and has written the final manuscript. P.G. has made substantial contributions to developing the arguments and contributed in the data collection for the article. All authors read and approved the final manuscript.

## Pre-publication history

The pre-publication history for this paper can be accessed here:

http://www.biomedcentral.com/1472-6963/13/229/prepub
